# Radiographic Projection Quality Score for the Knee (RPQS-Knee): Development and Reliability

**DOI:** 10.3390/diagnostics16172703

**Published:** 2026-08-25

**Authors:** Peter Melcher, Ralf Henkelmann, Salim Youssef, Anton Brehmer, Timm Denecke, Jeanette Henkelmann

**Affiliations:** 1 Department of Orthopedics and Trauma Surgery, Helios Klinik Leisnig, 04703 Leisnig, Germany; 2 Department for Orthopedics, Trauma and Plastic Surgery, University Hospital Leipzig, 04103 Leipzig, Germany; ralf.henkelmann@medizin.uni-leipzig.de (R.H.);; 3 Department for Diagnostic and Interventional Radiology, University Hospital Leipzig, Liebigstraße 20, 04103 Leipzig, Germanyjeanette.henkelmann@medizin.uni-leipzig.de (J.H.)

**Keywords:** knee radiography, projection quality, image quality, radiographic positioning, reliability, construct validity, musculoskeletal imaging, quality assurance

## Abstract

**Background/Objectives:** Accurate patient positioning is essential for diagnostic quality in conventional knee radiography. Although established anatomical landmarks are routinely used to judge projection quality, assessment remains qualitative and observer-dependent. We therefore developed and validated the Radiographic Projection Quality Score for the Knee (RPQS-Knee), a standardized scoring system for objective projection quality assessment. **Methods:** The RPQS-Knee was derived from established radiographic positioning criteria, anatomical reference landmarks, and predefined geometrically based measurement thresholds. The final score was evaluated in 153 consecutive knee radiographic examinations, independently assessed by one radiologist and two orthopedic surgeons. Inter rater reliability was assessed using quadratically weighted Gwet’s AC2, intra rater reliability using weighted Cohen’s κ, and construct validity by correlation with separately assigned diagnostic adequacy ratings. **Results:** The RPQS-Knee comprises four projection-related criteria assessing fibular-head superimposition, tibial plateau alignment, and femoral-condyle congruency. Inter rater reliability was excellent for the total score (AC2 = 0.93; 95% CI, 0.92–0.95) and good to excellent for all individual items (AC2 = 0.76–0.85). Intra rater reliability ranged from κ = 0.59 to 0.81. RPQS-Knee subscores correlated strongly with diagnostic adequacy ratings (ρ = −0.69 and −0.78; both *p* < 0.001), providing evidence supporting construct validity. **Conclusions:** The RPQS-Knee translates established radiographic positioning criteria into a standardized, reproducible instrument for objective assessment of projection quality. Its excellent reliability and evidence supporting construct validity suggest that it may facilitate standardized quality assessment in clinical practice, quality assurance, and research.

## 1. Introduction

Conventional radiography remains a cornerstone of musculoskeletal imaging and is among the most frequently performed diagnostic procedures worldwide [1,2]. Knee radiographs are routinely obtained for the evaluation of traumatic injuries, degenerative joint disease, malalignment, and postoperative follow-up, and therefore represent one of the most common examinations in daily clinical practice [3,4,5].

The diagnostic value of conventional radiography depends not only on image acquisition and exposure parameters, but also on accurate patient positioning and beam alignment [6,7,8]. Projectional deviations may alter the radiographic appearance of anatomical structures, affect the visualization of joint morphology, and complicate image interpretation [9,10,11]. Consequently, radiographic positioning has long been recognized as an important determinant of image quality, and established acquisition recommendations describe characteristic anatomical landmarks that indicate correct projection geometry [12,13].

For knee radiography, these landmarks include fibular-head superimposition on anteroposterior projections, congruency of the femoral condyles on lateral projections, and alignment of the tibial plateau. Such criteria are widely used during image acquisition and interpretation because they reflect rotational and angular positioning errors [14,15,16,17]. However, despite their routine use, these criteria are predominantly described qualitatively and are commonly evaluated using observer-dependent judgement rather than standardized measurement methods.

As a result, projection quality assessment remains difficult to standardize across observers, institutions, and studies. While radiographic positioning recommendations define desirable image characteristics, they do not provide a structured framework for quantifying the degree of deviation from an optimal projection. To date, no standardized and validated instrument is available for objective assessment of projection quality in conventional knee radiography.

The development of a reproducible scoring system may help address this methodological gap. By translating established anatomical landmarks and projection-related radiographic criteria into explicit scoring categories, projection quality can be evaluated using standardized and comparable measures. Such an approach may facilitate quality assessment in clinical practice, quality assurance initiatives, and research settings.

Therefore, the aim of this study was to develop and validate the Radiographic Projection Quality Score for the Knee (RPQS-Knee), a standardized instrument for the assessment of projection quality in conventional knee radiographs. The RPQS-Knee was developed from projection-related anatomical landmarks and geometrically derived criteria identified from the literature and established radiographic positioning principles. The score was designed to quantify the degree of deviation from an ideal radiographic projection using parameters assessable on routine anteroposterior and lateral knee radiographs.

We hypothesized that established projection-related anatomical landmarks visible on standard knee radiographs can be integrated into a structured scoring system that enables objective assessment of projection quality. Furthermore, we hypothesized that the RPQS-Knee would demonstrate good to excellent inter rater reliability and good intra rater reliability when applied by independent observers. Finally, we hypothesized that higher RPQS-Knee values would be associated with more favourable diagnostic adequacy ratings, providing evidence consistent with the construct validity of the RPQS-Knee. As an exploratory objective, we additionally evaluated whether the RPQS-Knee could be applied by less experienced raters following standardized instruction.

## 2. Materials and Methods

### 2.1. Study Design

This monocentric observational study was conducted to derive and validate the Radiographic Projection Quality Score for the Knee (RPQS-Knee), a standardized assessment tool for the objective evaluation of projection quality in conventional knee radiographs.

The study consisted of two major components: (1) development of the RPQS-Knee and (2) validation of the final score. The primary objective was to determine whether the RPQS-Knee provides a reproducible assessment of projection quality in routine knee radiography. Secondary objectives included assessment of intra rater reliability and evaluation of the relationship between RPQS-Knee values and perceived diagnostic adequacy. In addition, an exploratory analysis was performed to evaluate the applicability of the score among less experienced raters.

The study was approved by the local ethics committee (185/25-ek) and conducted in accordance with the Declaration of Helsinki. The requirement for informed consent was waived because only anonymized, routinely acquired radiographic data were analyzed and no study-related intervention was performed.

An a priori sample size calculation was performed using G*Power (version 3.1.9.7, Heinrich Heine University Düsseldorf, Düsseldorf, Germany). The calculation was based on the planned construct validity analysis, assessing the association between RPQS-Knee values and diagnostic adequacy ratings.

Assuming a two-sided significance level of α = 0.05, a statistical power of 80%, and a minimum relevant correlation coefficient of ρ = 0.25, the required sample size was 123 examinations. To account for potential incomplete ratings and to ensure adequate precision for the planned reliability analyses, 153 consecutive knee examinations were included.

The final study sample therefore exceeded the predefined minimum sample size requirement. The goal of this methodology is to investigate the reproducibility of an image-based score and explicitly not to identify the factors contributing to poor image quality.

### 2.2. Development of the RPQS-Knee

The development of the RPQS-Knee followed a structured multi-phase approach consisting of conceptual development, parameter selection based on anatomy and the literature, definition of measurement thresholds, feasibility and reproducibility assessment, and construction of the final scoring system.

Phase 1: Conceptual Development

The conceptual basis of the RPQS-Knee is that conventional radiographs represent projection images in which deviations from ideal patient positioning and X-ray beam alignment alter the appearance of anatomical structures. Rotational and angular positioning errors produce predictable changes in the radiographic appearance of osseous landmarks and joint surfaces. The RPQS-Knee was therefore designed to quantify deviation from an ideal radiographic projection using projection-dependent anatomical surrogate markers that can be assessed on routine radiographs.

An ideal projection was defined as a radiograph demonstrating optimal alignment of predefined anatomical reference structures according to established radiographic positioning standards.

Phase 2: Projection Quality Parameters Based on Anatomy and the Literature

The RPQS-Knee was developed using established radiographic positioning criteria and anatomical reference landmarks previously described in the musculoskeletal imaging and orthopedic literature. The objective was not to generate novel image features, but to integrate projection-dependent radiographic characteristics known to reflect rotational or angular deviations from an ideal knee radiographic projection.

Candidate parameters were selected according to three predefined criteria: (1) established relevance for radiographic positioning quality in the published literature, (2) reproducible visibility on routine anteroposterior and lateral knee radiographs, and (3) a plausible anatomical relationship to rotational or angular projection errors.

Posterior femoral-condyle congruency on lateral radiographs was included because the superimposition of the femoral condyles is internationally recognized as a fundamental quality criterion of a true lateral knee radiograph and has consistently been described as a surrogate marker of rotational alignment and projection accuracy [13,18].

Fibular-head superimposition on anteroposterior radiographs was selected based on previous investigations evaluating the influence of lower-limb rotation on knee radiographic appearance. The degree of overlap between the fibular head and the lateral tibial plateau varies systematically with rotational positioning and therefore provides an anatomically meaningful indicator of rotational projection error [19,20,21].

Double contour formation of the tibial plateau in both anteroposterior and lateral projections was selected as an indicator of angular misalignment between the tibial joint surface and the X-ray beam. Anatomical and morphometric studies have demonstrated that even small angular deviations result in measurable contour separation of the tibial plateau, making the magnitude of the projected double contour a suitable radiographic surrogate for angular positioning error [13,16,22].

The medial tibial plateau was selected as the anatomical reference structure because its dimensions and morphology have been extensively characterized in morphometric investigations, providing reproducible anatomical reference values for subsequent geometric modelling [16,23,24].

Foreign-object superimposition was additionally considered as a potential image quality parameter because external artefacts may impair image interpretation independently of projection quality. However, this parameter was not intended to assess projection geometry itself.

Thus, all candidate parameters were derived from previously described anatomical landmarks and established radiographic quality criteria, rather than from data-driven feature selection procedures.

Phase 3: Anatomically Derived Definition of Measurement Thresholds

Measurement thresholds for the double contour parameters were defined a priori, using anatomical dimensions reported in published morphometric studies of the tibial plateau. Thresholds were established before reliability analyses and were not derived from the study dataset.

Projected contour displacement resulting from angular deviation was estimated according to trigonometric principles. Assuming an approximately planar articular reference surface, projected displacement was calculated as the product of the anatomical reference dimension and the sine of the angular deviation.

The geometric model was intended to provide an approximate anatomical basis for ordinal categorization of projectional deviation, rather than to reconstruct the exact three-dimensional degree of malpositioning.

Published morphometric data describing the dimensions of the medial tibial plateau served as anatomical reference values. For the anteroposterior projection, a reference dimension of approximately 50 mm yielded projected contour separations of approximately 1.7 mm at 2° and 3.5 mm at 4° of angular deviation. For operationalization within the ordinal scoring system, these geometrically derived estimates were translated into pragmatic measurement thresholds of 1.5 mm and 3.5 mm for the anteroposterior projection and 1.0 mm and 2.0 mm for the lateral projection. These thresholds were intended to categorize the magnitude of radiographically visible projectional deviation rather than to provide exact patient-specific estimates of angular malpositioning. For the lateral projection, a reference dimension of approximately 29 mm yielded projected contour separations of approximately 1.0 mm at 2° and 2.0 mm at 4° [24,25,26,27].

These anatomically derived contour separations were subsequently used to define the ordinal score categories representing optimal, acceptable, and suboptimal projection quality. No threshold optimization based on study outcomes, reliability results, or diagnostic adequacy ratings was performed.

Phase 4: Feasibility and Reproducibility Assessment

Candidate parameters were subsequently evaluated regarding feasibility of assessment, rating variability, and reproducibility.

Foreign-object superimposition demonstrated negligible variability within the study population and therefore did not contribute to discrimination between examinations. Consequently, it was excluded from the final score and retained solely as an image quality flag.

A distance-based fibular alignment parameter demonstrated inferior reproducibility compared with the overlap-based fibular-head superimposition criterion and was therefore not retained in the final scoring system.

Item retention was based on predefined considerations of anatomical relevance, feasibility, sufficient rating variability, and reproducibility.

Phase 5: Construction of the Final RPQS-Knee

Following feasibility and reproducibility assessment, the final RPQS-Knee consisted of four projection-related items:

Fibular-head superimposition on anteroposterior radiographs;

Double contour of the medial tibial plateau on anteroposterior radiographs;

Posterior femoral-condyle congruency on lateral radiographs;

Double contour of the tibial plateau on lateral radiographs.

All items contributed equally to the total score.

Detailed operational scoring criteria and thresholds for all four RPQS-Knee components are provided in Supplementary Table S1.

**Figure 1:**
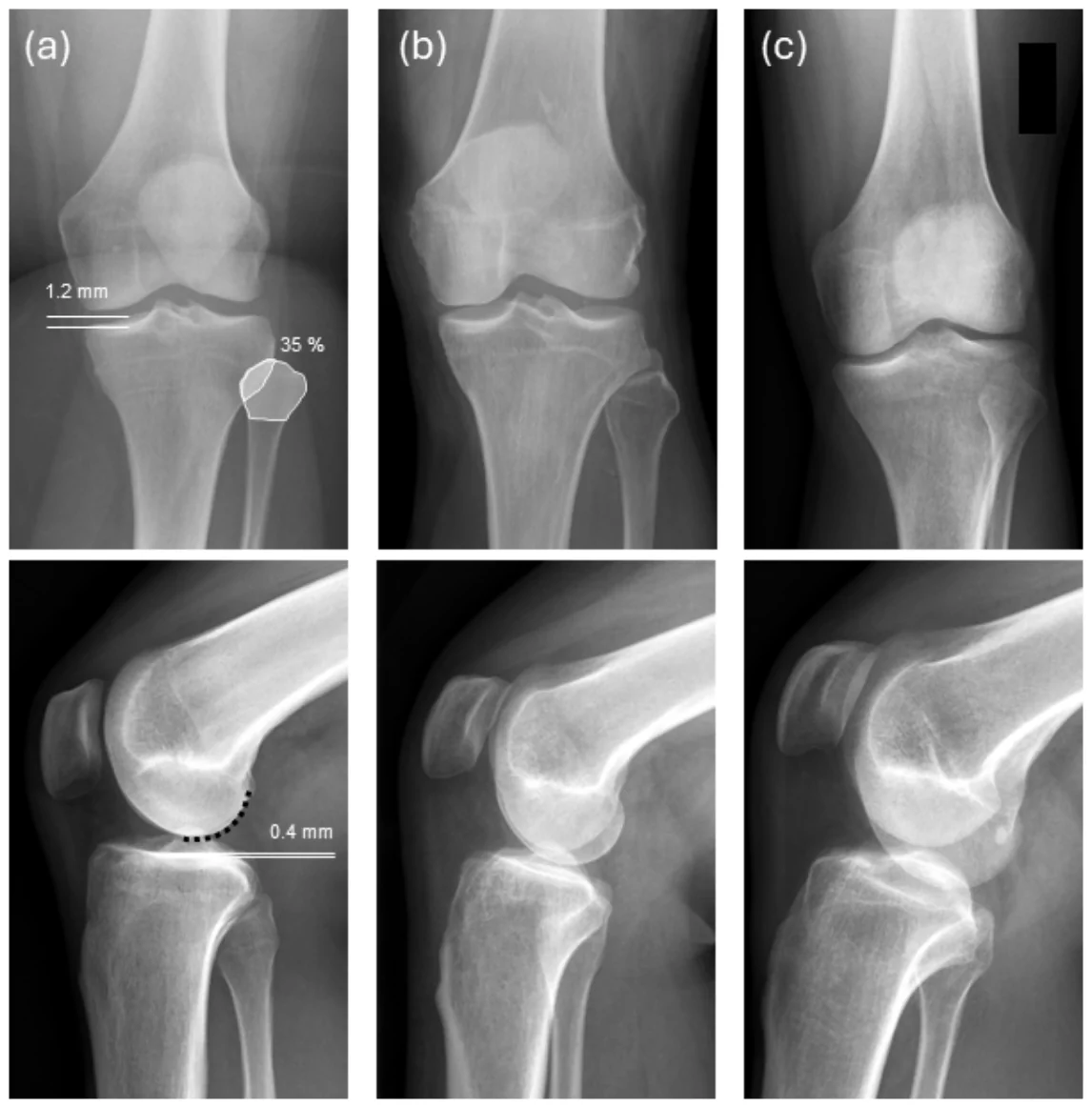
Representative application of the Radiographic Projection Quality Score for the Knee (RPQS-Knee). Representative anteroposterior (upper row) and lateral (lower row) knee radiographs demonstrating excellent (**a**), intermediate (**b**), and reduced (**c**) projection quality, corresponding to RPQS-Knee scores of 9, 7, and 5, respectively. The example with optimal projection quality (**a**) illustrates the measurement principles used for RPQS-Knee assessment, including fibular-head superimposition and medial tibial plateau double contour on the anteroposterior projection, as well as posterior femoral-condyle congruency and tibial plateau double contour on the lateral projection. The dotted contour in the lateral image highlights the posterior femoral-condyle contour used for assessment of condylar congruency. Complete scoring criteria are provided in Supplementary Table S1.

The RPQS-Knee was designed to quantify projection quality rather than directly estimate the exact degree of rotational or angular malpositioning. The selected parameters represent clinically applicable radiographic surrogates of projectional alignment that can be assessed without complex image processing or three-dimensional reconstruction. Representative examples of all scoring categories are provided in Figure 1.

### 2.3. Radiographic Dataset and Rating Procedure

A total of 153 consecutive knee examinations were retrospectively included. The examinations originated from patients presenting to the emergency department, outpatient clinics, and inpatient wards of a tertiary referral centre. Each examination consisted of one anteroposterior and one lateral radiograph, resulting in 306 radiographic images.

Radiographs of patients younger than 18 years and radiographs demonstrating grossly displaced fractures were excluded. Prior to assessment, all images were anonymized. Patient-identifying metadata, annotations, and acquisition-related identifiers were removed before re-importing into the image-viewing system. Radiographs containing arthroplasty or osteosynthesis implants were excluded. Osteoarthritis was not an exclusion criterion and was not systematically graded. One postoperative examination containing a localized bone-cement spacer following treatment of a bone cyst was retained because the predefined anatomical landmarks remained assessable.

The analysis dataset consisted of anonymized radiographic data. Detailed demographic and clinical variables, including age, sex, BMI, joint flexibility, and clinical alignment, were not retained and were therefore not available for subgroup or multivariable analyses. Axial deformity was not systematically assessed because dedicated whole-leg radiographs were not available for all examinations.

For the primary analyses, 152 complete cases were available. One examination was excluded from score-level analyses because of incomplete ratings. Analyses involving individual score items were performed using complete cases for the respective variable.

Image assessment was performed independently by three experienced clinicians, consisting of one radiologist and two orthopedic surgeons. Before evaluation, all raters received standardized training regarding the RPQS-Knee. Training included explanation of all score criteria and assessment of representative example radiographs.

All image assessments were performed using the institutional picture archiving and communication system (PACS; IDS7, Sectra AB, Linköping, Sweden). Distance and area measurements were obtained using the calibrated digital measurement tools integrated into the PACS software.

In addition to RPQS-Knee assessment, each rater separately assigned a global diagnostic adequacy rating using a six-point ordinal scale, with 1 indicating excellent diagnostic quality and 6 indicating non-diagnostic image quality. The diagnostic adequacy rating was not incorporated into the RPQS-Knee calculation and was used solely as a conceptually related global rating for construct validity assessment. To assess intra rater reliability, all radiographs were reassessed after an interval of three months. Prior to the second assessment, image order was randomized to minimize recall bias. The second rating round was prespecified as the primary dataset for inter rater reliability analyses, whereas agreement between the first and second rating rounds was used to assess intra rater reliability.

To minimize potential cross-score influence, the global diagnostic adequacy rating was assigned before assessment of the individual RPQS-Knee components.

### 2.4. Outcome Measures and Statistical Analysis

The primary outcome was the inter rater reliability of the total RPQS-Knee score.

Secondary outcomes included inter rater reliability of individual score items and subscores, intra rater reliability of all score components, and the association between RPQS-Knee values and global diagnostic adequacy ratings. An exploratory outcome was agreement between experienced and novice raters.

Descriptive statistics were used to summarize distributions of RPQS-Knee items, subscores, total scores, and diagnostic adequacy ratings. Ordinal variables were summarized using frequencies and percentages, whereas composite scores were summarized using means, medians, and frequency distributions.

Inter rater reliability was assessed using Gwet’s AC2 with quadratic weighting [28,29]. Reliability analyses were performed for each RPQS-Knee item, the anteroposterior subscore, the lateral subscore, the total RPQS-Knee score, and the diagnostic adequacy ratings.

Intra rater reliability was assessed by comparing the first and second rating rounds using weighted Cohen’s kappa with quadratic weighting [30].

Construct validity was evaluated by assessing the association between RPQS-Knee values and diagnostic adequacy ratings using Spearman’s rank correlation coefficient. Because higher RPQS-Knee values indicate superior projection quality, whereas lower diagnostic adequacy ratings indicate better perceived image quality, negative correlations were expected.

Statistical analyses were performed using R version 4.5.2 (R Foundation for Statistical Computing, Vienna, Austria) and IBM SPSS Statistics version 31 (IBM Corp., Armonk, NY, USA). A *p*-value < 0.05 was considered statistically significant.

### 2.5. Exploratory Analysis of Novice Raters

To explore whether the RPQS-Knee can be applied by less experienced observers, two senior medical students independently assessed all radiographs after receiving the same training and instructions as the experienced raters.

Agreement between novice consensus ratings and expert consensus ratings was evaluated for the anteroposterior subscore, lateral subscore, and diagnostic adequacy rating using weighted Cohen’s kappa with quadratic weighting. This analysis was considered exploratory.

### 2.6. Use of Generative Artificial Intelligence

Generative artificial intelligence was used during manuscript preparation to support language editing, including the correction of grammar and spelling, the improvement of sentence structure and readability, and suggestions to improve textual clarity and flow. The AI tool was not used to generate or modify study data or to make autonomous scientific or clinical interpretations. All AI-assisted text was critically reviewed, revised where necessary, and approved by the authors, who take full responsibility for the scientific content of the manuscript.

## 3. Results

### 3.1. Study Sample

A total of 153 consecutive knee examinations consisting of one anteroposterior and one lateral radiograph per examination were included, resulting in 306 radiographic images. No examination demonstrated foreign-object superimposition affecting radiographic assessment.

### 3.2. Feasibility and Reproducibility Assessment of Candidate Parameters

All candidate parameters were assessable on routine knee radiographs.

Foreign-object superimposition showed no relevant variability within the study sample. Consequently, this parameter was not included in the final RPQS-Knee calculation and was retained only as an image quality flag.

The distance-based fibular alignment criterion demonstrated lower reproducibility than the overlap-based fibular-head superimposition criterion. Inter rater reliability for the distance-based criterion was AC2 = 0.481 (95% CI: 0.37–0.60). Corresponding intra rater reliability values were κw = 0.36, 0.29, and 0.421 for the three experienced raters.

The final RPQS-Knee consisted of four projection-related items: fibular-head superimposition, medial tibial plateau double contour, posterior femoral-condyle congruency, and tibial plateau double contour.

### 3.3. Distribution of Final RPQS-Knee Scores

In the second rating round, pooled ratings across the three experienced raters demonstrated variability across all final RPQS-Knee items.

Fibular-head superimposition was rated as 1 point in 35.7%, 2 points in 41.4%, and 3 points in 22.8% of assessments. The medial tibial plateau double contour was rated as 1 point in 18.6%, 2 points in 53.1%, and 3 points in 28.3%.

Posterior femoral-condyle congruency was rated as 1 point in 60.0%, 2 points in 33.4%, and 3 points in 6.6%. The tibial plateau double contour was rated as 1 point in 46.7%, 2 points in 25.0%, and 3 points in 28.3%.

The anteroposterior subscore showed a mean value of 4.0 points (median 4), whereas the lateral subscore showed a mean value of 3.3 points (median 3). The total RPQS-Knee score showed a mean value of 7.2 points and a median value of 7 points.

### 3.4. Inter Rater Reliability

Inter rater reliability was assessed using Gwet’s AC2 with quadratic weighting, based on the second rating round (Table 1).

**Figure 2:**
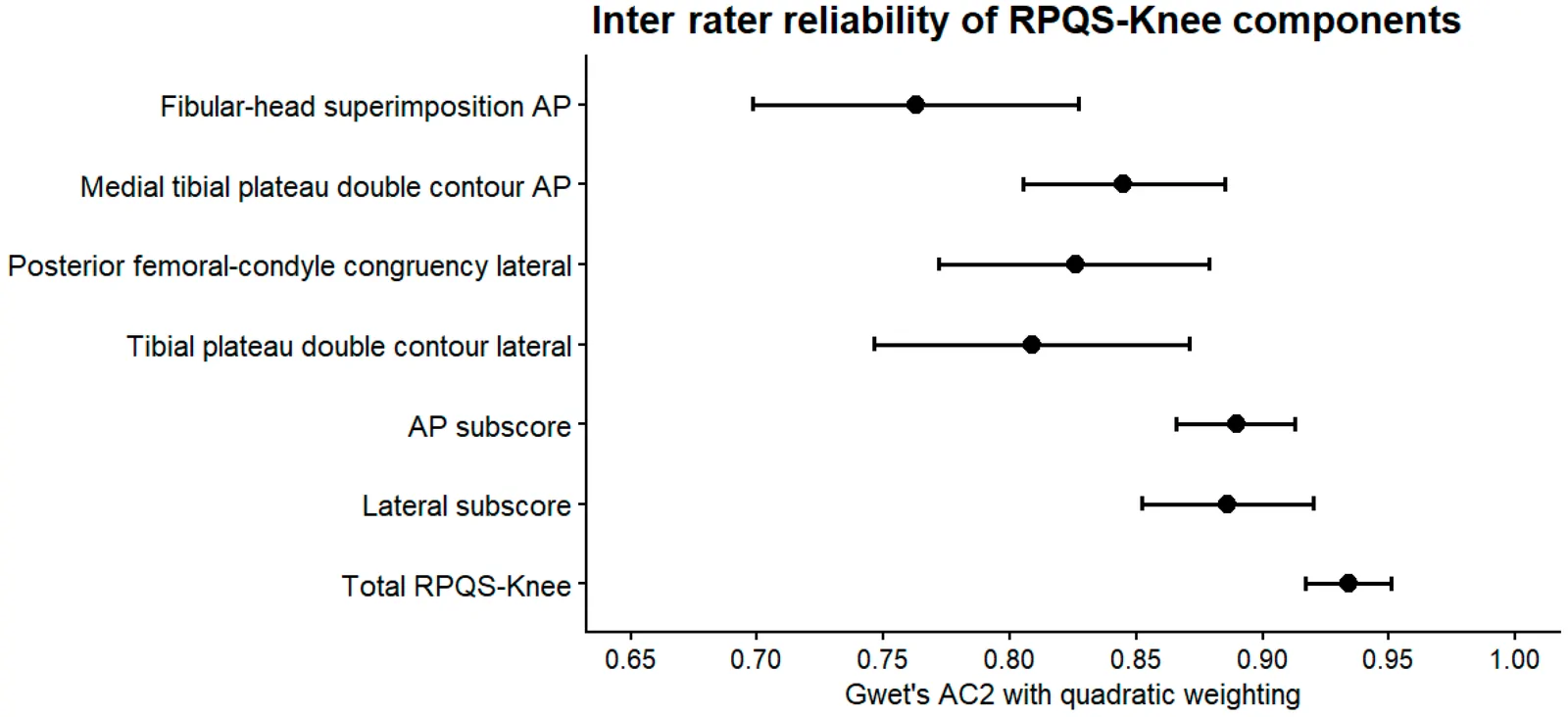
Inter rater reliability of RPQS-Knee components.

For the individual RPQS-Knee items, AC2 values ranged from 0.76 to 0.85 for the anteroposterior projection and from 0.81 to 0.83 for the lateral projection. For the composite scores, AC2 was 0.89 for the anteroposterior subscore, 0.89 for the lateral subscore, and 0.93 for the total RPQS-Knee score. Inter rater reliability estimates and corresponding confidence intervals are summarized in Figure 2.

Inter rater reliability of the diagnostic adequacy rating was 0.88 for the anteroposterior projection and 0.89 for the lateral projection.

### 3.5. Intra Rater Reliability

Intra rater reliability was assessed by comparing the first and second rating rounds using weighted Cohen’s kappa with quadratic weighting (Table 2).

For fibular head superimposition, κw values ranged from 0.59 to 0.69. For the medial tibial plateau double contour, κw values ranged from 0.61 to 0.77. For posterior femoral-condyle congruency, κw values ranged from 0.63 to 0.75. For the lateral tibial plateau double contour, κw values ranged from 0.79 to 0.81.

For the diagnostic adequacy rating, κw values ranged from 0.39 to 0.58 for the anteroposterior projection and from 0.67 to 0.87 for the lateral projection.

### 3.6. Construct Validity

Spearman correlation analyses demonstrated significant associations between RPQS-Knee subscores and diagnostic adequacy ratings (Table 3).

**Figure 3:**
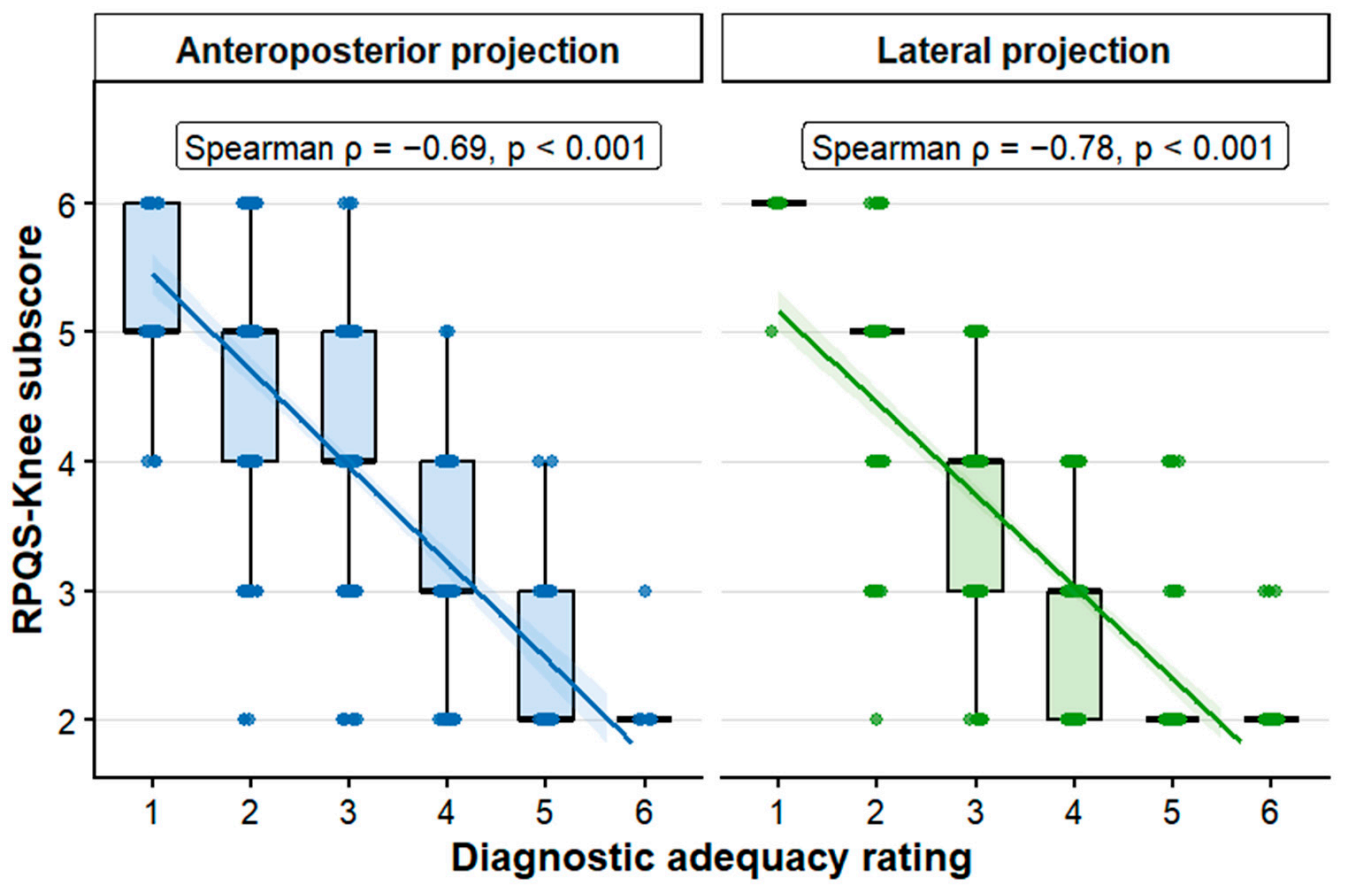
RPQS-Knee subscores according to diagnostic adequacy ratings. Boxplots show median, interquartile range, and individual observations with jittered points. Linear trend lines with 95% confidence bands are shown for visualization. Spearman’s rank correlation was used for statistical analysis. Lower diagnostic adequacy ratings indicate better perceived diagnostic quality, whereas higher RPQS-Knee values indicate better projection quality.

For pooled ratings across all experienced raters, the anteroposterior subscore correlated with the anteroposterior diagnostic adequacy rating at ρ = −0.69, whereas the lateral subscore correlated with the lateral diagnostic adequacy rating at ρ = −0.78 (both *p* < 0.001). The relationship between RPQS-Knee subscores and diagnostic adequacy ratings is illustrated in Figure 3.

At the individual rater level, correlations ranged from −0.61 to −0.87 for the anteroposterior projection and from −0.74 to −0.81 for the lateral projection.

### 3.7. Exploratory Applicability Assessment

Agreement between novice and experienced raters was κw = 0.71 (95% CI: 0.64–0.78; *p* < 0.001) for the anteroposterior subscore and κw = 0.74 (95% CI: 0.67–0.81; *p* < 0.001) for the lateral subscore.

For diagnostic adequacy ratings, agreement was κw = 0.65 (95% CI: 0.57–0.73; *p* < 0.001) for the anteroposterior projection and κw = 0.60 (95% CI: 0.52–0.69; *p* < 0.001) for the lateral projection.

## 4. Discussion

### 4.1. Principal Findings

This study describes the development and evaluation of the Radiographic Projection Quality Score for the Knee (RPQS-Knee), a standardized instrument designed to assess projection quality in conventional knee radiography using anatomically defined radiographic landmarks. The final score consists of four projection-related criteria derived from established radiographic positioning principles and anatomical reference structures.

The RPQS-Knee demonstrated high reproducibility across independent observers, with excellent agreement observed for the composite score and consistently good agreement across all retained score components. In addition, RPQS-Knee subscores showed strong associations with separately assigned diagnostic adequacy ratings. Together, these findings indicate that the score can be applied reproducibly and captures image characteristics that are relevant to overall radiographic quality assessment.

An additional finding of this study was that not all anatomically plausible parameters demonstrated sufficient reproducibility for routine application. The distance-based fibular alignment criterion showed substantially lower agreement than the overlap-based fibular-head criterion and was therefore excluded from the final score. This observation highlights the importance of combining anatomical rationale with empirical reproducibility testing during score development.

### 4.2. Reliability and Construct Validity

A principal objective of the present study was to develop a reproducible method for evaluating radiographic projection quality. The results indicate that the RPQS-Knee can be applied consistently across independent observers under routine clinical conditions.

The choice of Gwet’s AC2 as the primary inter rater agreement coefficient was intentional. Agreement measures based on kappa statistics may be influenced by prevalence effects and imbalanced marginal distributions, particularly when ordinal rating scales contain categories that occur infrequently. Because the RPQS-Knee consists of ordinal criteria with non-uniform category distributions, Gwet’s AC2 was considered the most appropriate measure for evaluating observer agreement [28,29].

An important observation was that agreement was higher for the composite score than for the individual score items. It should be noted that intra rater reliability was lower for some individual components than the inter rater reliability of the composite score. For fibular-head superimposition, for example, κw ranged from 0.59 to 0.69. Thus, individual landmarks may be more susceptible to observer-dependent measurement variability than the combined score. This finding is consistent with the conceptual design of the RPQS-Knee. Individual radiographic landmarks may be influenced by anatomical variation, image quality, or observer interpretation. By integrating several projection-related criteria into a composite score, the influence of variability associated with any single measurement is reduced, resulting in a more stable overall assessment. The excellent agreement observed for the total RPQS-Knee supports this approach and suggests that projection quality can be assessed more reliably through a structured combination of anatomical criteria than through isolated evaluation of individual radiographic features.

The lower reproducibility observed for the distance-based fibular criterion illustrates that anatomical plausibility alone is insufficient for inclusion in a practical scoring system. The final RPQS-Knee therefore reflects a balance between anatomical relevance and reproducible application in routine radiographs.

To our knowledge, no objective reference standard currently exists for quantifying radiographic projection quality in conventional knee radiography. Unlike technical image quality parameters such as exposure or noise, projection quality reflects the geometric relationship between anatomy, patient positioning, and beam alignment, and cannot be directly measured using an established external standard. Consequently, construct validation of projection quality instruments may rely on comparison with conceptually related measures that reflect the underlying construct [12,13].

Construct validity was evaluated through comparison with separately assigned diagnostic adequacy ratings. The strong associations observed between RPQS-Knee subscores and diagnostic adequacy ratings support the assumption that the score captures image characteristics that are relevant to overall radiographic quality assessment. At the same time, the absence of perfect correlations is consistent with the conceptual distinction between both measures. Whereas the RPQS-Knee specifically quantifies projection quality, diagnostic adequacy ratings represent a broader assessment of image suitability and may additionally reflect factors such as exposure, contrast, noise, artefacts, and observer-specific diagnostic expectations.

Because both measures were assigned by the same raters during the same evaluation session, residual cross-score bias and conceptual overlap cannot be excluded.

An additional strength of the present study is that score development and validation were performed using radiographs acquired during routine clinical care rather than highly selected teaching datasets. Examinations originated from emergency, outpatient, and inpatient settings, and therefore encompassed the variability typically encountered in daily practice. This increases confidence that the observed agreement estimates are relevant to real-world clinical application.

### 4.3. Implications for Standardized Radiographic Quality Assessment

The primary contribution of the RPQS-Knee is not the introduction of new radiographic criteria but the standardization of criteria that are already used in routine practice. Although projection quality is routinely considered during image interpretation, assessment is frequently based on qualitative judgement and individual experience. As a result, projection quality may be described inconsistently across observers, institutions, and studies.

The RPQS-Knee provides a structured framework for quantifying projection quality using predefined anatomical criteria and explicit scoring categories. By transforming established positioning characteristics into a reproducible measurement instrument, the score enables projection quality to be documented and evaluated in a standardized manner. This may facilitate more objective comparison of radiographs within clinical practice, quality assurance initiatives, and future research studies.

Importantly, the present study was designed to evaluate measurement properties of the RPQS-Knee rather than its clinical consequences. The score should therefore be regarded as a projection quality assessment instrument and not as a decision tool for image acceptance, repeat acquisition, or diagnostic interpretation. Whether specific RPQS-Knee values are associated with clinically relevant outcomes remains a separate question requiring dedicated investigation [13,17,18].

### 4.4. Limitations and Future Directions

Several limitations should be considered. First, this was a single-centre study, and external validation in independent institutions is required before broader generalization can be recommended. Second, the RPQS-Knee was developed specifically for conventional knee radiographs and cannot automatically be transferred to other anatomical regions. Third, construct validation was performed using diagnostic adequacy ratings rather than an objective external reference standard. Because both measures were assigned by the same raters during the same evaluation session, residual cross-score bias and conceptual overlap cannot be excluded. Finally, the exploratory applicability assessment included only two novice raters and was not designed to evaluate training requirements or performance across different professional groups.

Detailed demographic variables such as age distribution and sex were not retained in the anonymized analysis dataset. Since this is a retrospective analysis of X-ray images taken at a tertiary care facility, conclusions can only be drawn regarding this specific patient population. Potential differences in RPQS-Knee performance related to patient-specific anatomical or clinical characteristics could therefore not be investigated.

As with conventional radiography in general, three-dimensional positioning abnormalities cannot be reconstructed with certainty from a single two-dimensional projection. Fixed millimetre thresholds are based on population-derived anatomical reference dimensions and therefore cannot account for the full range of individual variation in tibial size and morphology. Accordingly, the thresholds should be interpreted as pragmatic screening categories rather than patient-specific estimates of angular deviation.

Manual measurement of small contour separations may be affected by image resolution, the definition of cortical margins, and the selection of measurement points, particularly for measurements close to category boundaries.

The exploratory novice rater findings should be regarded as hypothesis-generating and cannot be generalized to inexperienced users or to other professional groups.

Future studies should evaluate the performance of the RPQS-Knee in external cohorts and investigate whether RPQS-Knee values can support clinically relevant decisions regarding image acceptability and repeat image acquisition. In addition, the methodological framework presented here may serve as a basis for the development of projection quality assessment tools for other anatomical regions.

## 5. Conclusions

The RPQS-Knee is a standardized radiographic projection quality assessment tool derived from anatomically defined radiographic landmarks. The score demonstrated good to excellent reliability across individual items and excellent reliability for the composite score. Strong associations with separately assigned diagnostic adequacy ratings provide evidence supporting the construct validity of the RPQS-Knee. The RPQS-Knee provides a reproducible framework for standardized assessment of projection quality in conventional knee radiography.

## Figures and Tables

**Table 1:** Inter rater reliability of RPQS-Knee items, subscores, total score, and diagnostic adequacy ratings.

Parameter	*N*	Gwet’s AC2	SE	95% CI	*p*-Value
Fibular-head superimposition AP	152	0.76	0.03	0.70–0.83	<0.001
Medial tibial plateau double contour AP	152	0.85	0.02	0.81–0.89	<0.001
Posterior femoral-condyle congruency lateral	151	0.83	0.03	0.77–0.88	<0.001
Tibial plateau double contour lateral	152	0.81	0.03	0.75–0.87	<0.001
AP subscore	152	0.89	0.01	0.87–0.91	<0.001
Lateral subscore	151	0.89	0.02	0.85–0.92	<0.001
Total RPQS-Knee	151	0.93	0.01	0.92–0.95	<0.001
Diagnostic adequacy rating AP	152	0.88	0.01	0.86–0.90	<0.001
Diagnostic adequacy rating lateral	152	0.89	0.01	0.86–0.91	<0.001

**Table 2:** Intra rater reliability of RPQS-Knee items and diagnostic adequacy ratings.

Criterion	Rater 1 κw	Rater 2 κw	Rater 3 κw
Fibular-head superimposition AP	0.63	0.69	0.59
Distance-based fibular criterion AP	0.36	0.29	0.42
Medial tibial plateau double contour AP	0.61	0.77	0.71
Diagnostic adequacy rating AP	0.45	0.58	0.39
Tibial plateau double contour lateral	0.79	0.81	0.81
Posterior femoral-condyle congruency lateral	0.65	0.75	0.63
Diagnostic adequacy rating lateral	0.67	0.87	0.80

**Table 3:** Spearman correlations between RPQS-Knee subscores and diagnostic adequacy ratings.

Rater	Projection	*N*	Spearman ρ
Rater 1	AP	152	−0.76
Rater 1	Lateral	152	−0.81
Rater 2	AP	152	−0.61
Rater 2	Lateral	151	−0.74
Rater 3	AP	152	−0.87
Rater 3	Lateral	152	−0.81
Pooled ratings	AP	456	−0.69
Pooled ratings	Lateral	455	−0.78

All correlations *p* < 0.001.

## Data Availability

Anonymized data supporting the findings of this study are available from the corresponding author upon reasonable request, subject to applicable ethical and data-protection requirements.

## Supplementary Materials

The following supporting information can be downloaded at: https://www.mdpi.com/article/10.3390/diagnostics16172703/s1, Table S1: Radiographic Projection Quality Score for the Knee (RPQS-Knee): scoring criteria and operational thresholds.
