# Supplementary material for: Radiographic Projection Quality Score for the Knee (RPQS-Knee): Development and Reliability
Source: Diagnostics (Basel). 2026 Aug 25;16(17):2703. doi: 10.3390/diagnostics16172703 (PMC13564488; doi:10.3390/diagnostics16172703)
Supplement: Supplementary file 1 [file diagnostics-16-02703-s001.zip › diagnostics-4483485-supplementary.pdf]

Supplementary Table S1. Radiographic Projection Quality Score for the Knee (RPQS-Knee): scoring criteria and operational thresholds

Projection	Criterion	3 points	2 points	1 point
**AP**	Fibular-head superimposition	31–37%	20–<31% or >37–45%	<20% or >45%
	Medial tibial plateau double contour	<1.5 mm	1.5–3.5 mm	>3.5 mm
**Lateral**	Posterior femoral-condyle congruency	Complete superimposition	Near-complete superimposition	Inadequate superimposition
	Tibial plateau double contour	<1.0 mm	1.0–2.0 mm	>2.0 mm

AP subscore: 2–6 points; **lateral subscore:** 2–6 points; **total RPQS-Knee score:** 4–12 points. **Higher scores indicate better projection quality.**

Abbreviations: AP, anteroposterior; RPQS-Knee, Radiographic Projection Quality Score for the Knee.
